# Dioxinodehydroeckol Enhances the Differentiation of Osteoblasts by Regulating the Expression of Phospho-Smad1/5/8

**DOI:** 10.3390/md14090168

**Published:** 2016-09-14

**Authors:** Byul-Nim Ahn, Fatih Karadeniz, Chang-Suk Kong, Ki-Ho Nam, Mi-Soon Jang, Youngwan Seo, Han Seong Kim

**Affiliations:** 1 Department of Organic Material Science and Engineering, Pusan National University, Busan 46241, Korea; icetwig@naver.com; 2 Marine Biotechnology Center for Pharmaceuticals and Foods, Silla University, Busan 46958, Korea; karadenizf@outlook.com (F.K.); cskong@silla.ac.kr (C.-S.K.); 3 Department of Food and Nutrition, College of Medical and Life Sciences, Silla University, Busan 46958, Korea; 4 Food Safety and Processing Research Division, National Institute of Fisheries Science, Busan 46083, Korea; dennis011@korea.kr (K.-H.N.); suni1@korea.kr (M.-S.J.); 5 Division of Marine Bioscience, College of Ocean Science and Technology, Korea Maritime and Ocean University, Busan 49112, Korea; ywseo@kmou.ac.kr; 6 Department of Convergence Study on the Ocean Science and Technology, Ocean Science and Technology School, Korea Maritime and Ocean University, Busan 49112, Korea

**Keywords:** BMP-2, dioxinodehydroeckol, *Ecklonia cava*, osteoblastogenesis, Smad

## Abstract

Lack of bone formation-related health problems are a major problem for the aging population in the modern world. As a part of the ongoing trend of developing natural substances that attenuate osteoporotic bone loss conditions, dioxinodehydroeckol (DHE) from edible brown alga *Ecklonia cava* was tested for its effects on osteoblastogenic differentiation in MC3T3-E1 pre-osteoblasts. DHE was observed to successfully enhance osteoblast differentiation, as indicated by elevated cell proliferation, alkaline phosphatase activity, intracellular cell mineralization, along with raised levels of osteoblastogenesis indicators at the concentration of 20 μM. Results suggested a possible intervening of DHE on the bone morphogenetic protein (BMP) signaling pathway, according to elevated protein levels of BMP-2, collagen-I, and Smads. In addition, the presence of DHE was also able to raise the phosphorylated extracellular signal–regulated kinase (ERK) and c-Jun N-terminal kinase (JNK) levels which are also activated by the BMP signaling pathway. In conclusion, DHE is suggested to be a potential bioactive compound against bone loss that could enhance osteoblastogenesis with a suggested BMP pathway interaction.

## 1. Introduction

In today’s world, bone-related issues are considered to be one of the major causes of low quality of life, morbidity, and mortality [1]. Osteoporosis, which is associated with an imbalance in bone mass, is one of the most common bone-related disorders. Patients above the age of 50 suffering from bone fractures are predominantly diagnosed with osteoporosis [2]. There are several treatment options for osteoporosis through drugs or functional foods; namely, estrogen therapy, bisphosphonates, vitamin D analogues, and receptor activator of nuclear factor-κB ligand (RANKL) inhibition [3,4,5]. The common idea behind osteoporosis treatment is to promote bone tissue formation by osteoblast differentiation while preventing possible causes of bone volume loss. Elevated differentiation into osteocytes helps to relieve deteriorated bone mass balance [6]. However, the close relation of the onset of osteoblastogenesis with obesity and diabetes is a major concern for the well-being of aged patients who also suffer from metabolic syndromes [7,8]. Towards a more efficient control of osteoporosis, natural sources and bioactive substances are at the center of attention for novel treatment options with fewer expected side effects. Several studies have reported marine-based natural compounds that enhance osteoblastogenesis in vitro [9,10].

For centuries, *Ecklonia cava* (Laminariaceae) has been present as an abundant edible marine algae and has been consumed as a kind of seasoned delicacy in the coastal areas of Asian countries, including Korea and Japan [11,12,13]. Moreover, it has been studied as a valuable source of nutraceuticals, due to its broad range of evidential biological activity [14,15,16,17]. *E. cava* is shown to contain various bioactive substances, including phlorotannins, which have been hailed as compounds with possible pharmacological value. Phlorotannins are an oligomeric form of phloroglucinol [18], and have been revealed to have the following properties: antioxidant [19], antibacterial [20], anti-inflammation [21], anti-allergy [22], anti-matrix metalloproteinase (MMP) [23], and apoptosis induction [17]. Therefore, in the present study, as part of our continuing research of nutraceutical components from *E. cava*, the in vitro osteoblastogenesis-promoting activity of dioxinodehydroeckol (DHE) was evaluated through the investigation of the responsibility for osteoblastogenesis-inducing action in pre-osteoblasts.

## 2. Results

**Figure 1:**
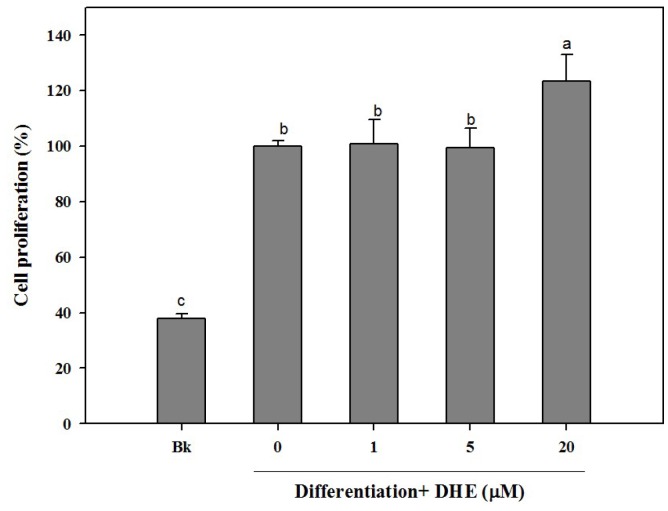
Effect of dioxinodehydroeckol (DHE) on the cell proliferation of differentiating MC3T3-E1 pre-osteoblasts. Values are means ± SD (*n* = 3). ^a–c^ Means with different letters are significantly different (*p* < 0.05) by Duncan’s multiple range test. Bk: Non-differentiated blank group.

Different concentrations (1, 5, and 20 μM) of DHE were introduced to cell culture medium during the differentiation process. As a part of osteogenic differentiation, MC3T3-E1 cells were observed to show an increasing proliferation trend. In connection, the effect of DHE on the cell proliferation of differentiating pre-osteoblasts was assessed. Similar to untreated control cells, the presence of DHE also increased the proliferation of the osteoblasts (Figure 1). DHE was able to elevate the proliferation only in the highest treatment concentration (20 μM). Treatment of 1 and 5 μM samples was not able to significantly elevate the cell proliferation of differentiating cells compared to untreated control cells, whereas the non-differentiated blank cell count was only 39% of control cells (Figure 1). However, at the concentration of 20 μM, DHE showed a notable proliferation enhancement effect, raising the cell proliferation by 24% compared to untreated differentiated control cells, which were referred to as 100%.

**Figure 2:**
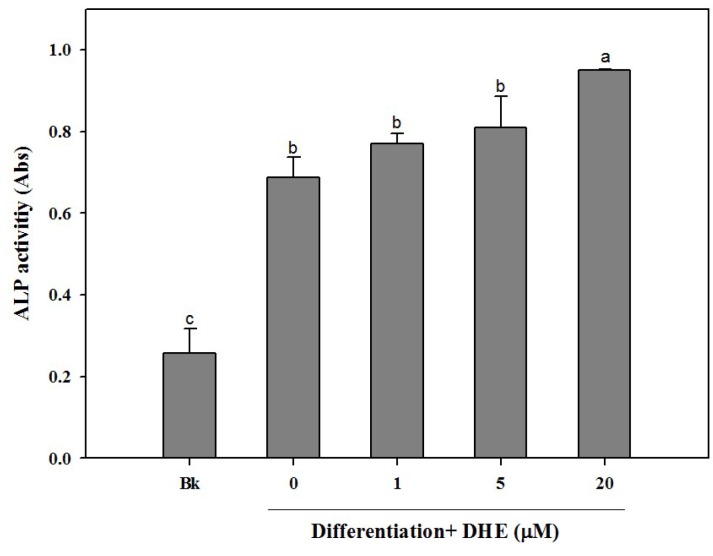
Effect of DHE on cellular alkaline phosphatase (ALP) activity in differentiated MC3T3-E1 cells. Values are means ± SD (*n* = 3). ^a–c^ Means with different letters are significantly different (*p* < 0.05) by Duncan’s multiple range test. Bk: Non-differentiated blank group.

Following the confirmation of the non-toxic presence of DHE in pre-osteoblasts, the possible osteoblastogenesis enhancing effect was also tested by an alkaline phosphatase (ALP) activity assay. Elevated ALP activity is evidently related to increased bone formation in osteoblasts. In a similar fashion to cell proliferation assay results, DHE was able to elevate the ALP activity in differentiating osteoblasts (Figure 2). DHE was observed to increase ALP activity to a level 33% higher than that of untreated differentiated control cells at the concentration of 20 μM. Likewise, 1 and 5 μM DHE treatments enhanced the ALP activity by 7% and 11% compared to control, respectively.

**Figure 3:**
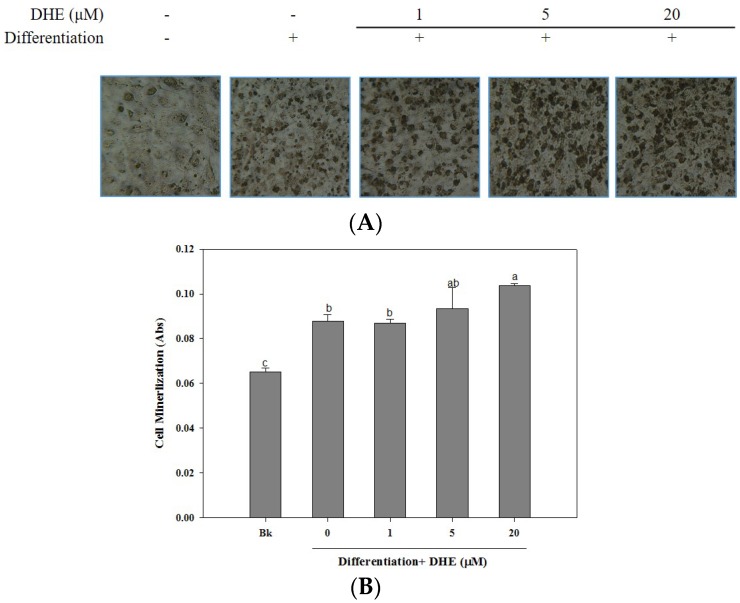
Effect of DHE on cellular mineralization in MC3T3-E1 cells presented as (**A**) alizarin red-stained cell images and (**B**) absorbance values of eluted dye retained in the cells. Values are means ± SD (*n* = 3). ^a–c^ Means with different letters are significantly different (*p* < 0.05) by Duncan’s multiple range test. Bk: Non-differentiated blank group.

The osteoblastogenesis-enhancing activity of DHE was also confirmed by the assessment of cellular mineralization through the Alizarin Red staining of intracellular calcification. As shown in Figure 3, the presence of DHE at a concentration of 20 μM significantly increased the intracellular calcium levels of differentiated osteoblasts, which might be linked to enhanced osteoblastogenesis. DHE-induced enhancement of bone formation through elevated cell mineralization was suggested by staining the intracellular calcification. Cell images clearly indicated that the presence of DHE increased the intracellular calcification suggested by stained calcium in a slight dose-dependent manner (Figure 3).

The mineralization of MC3T3-E1 cells increased by 28% during differentiation into osteoblasts (untreated differentiated control cells compared to non-differentiated blank cells). The presence of DHE increased intracellular calcification by 38%, 41%, and 54% at concentrations of 1, 5, and 20 μM, respectively. Treatment with 1 μM DHE did not show any significant enhancement, although 20 μM DHE notably promoted intracellular mineralization.

**Figure 4:**
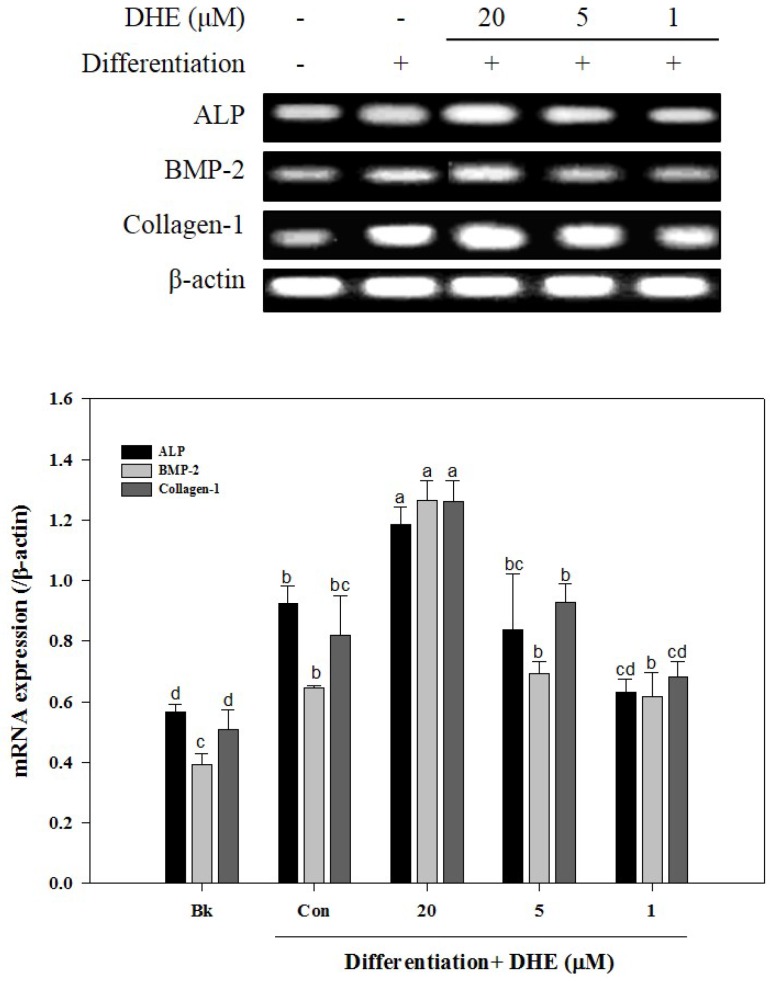
Effect of DHE on the mRNA expression levels of key osteoblastogenesis markers in MC3T3-E1. Values are means ± SD (*n* = 3). ^a–d^ Means with different letters are significantly different (*p* < 0.05) by Duncan’s multiple range test. Bk: Non-differentiated blank group; Con: Differentiated non-treated control group; ALP: alkaline phosphatase; BMP: bone morphogenic protein.

**Figure 5:**
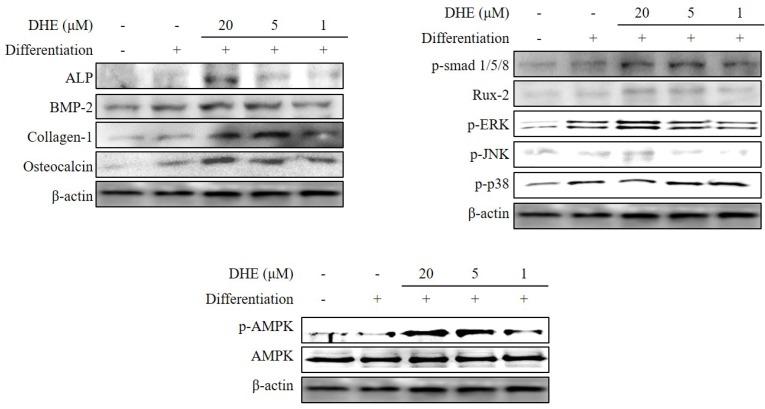
Effect of DHE on protein levels of key osteoblastogenesis pathway molecules in MC3T3-E1 cells. ALP: Alkaline phosphatase; BMP: bone morphogenic protein; p-smad: phosphorylated sma/mad homolog protein; Rux: runt-related transcription factor; p-ERK: phosphorylated extracellular signal–regulated kinase; p-JNK: phosphorylated c-Jun N-terminal kinase; p-p38: phosphorylated p38 protein; AMPK: 5′ AMP-activated protein kinase; p-AMPK: phosphorylated 5′ AMP-activated protein kinase.

After confirming that DHE isolated from *E. cava* affects differentiated osteoblasts by enhancing bone formation, a reverse transcription-polymerase chain reaction (RT-PCR) assay was carried out in order to suggest a possible pathway on which DHE acts. During osteogenic differentiation, MC3T3-E1 pre-osteoblasts were treated with DHE at the concentrations 1, 5, and 20 μM. Following full maturation into osteoblasts, mRNA expressions of key osteogenic markers (namely ALP, bone morphogenetic protein (BMP)-2, and collagen-I) were quantified by RT-PCR. DHE significantly enhanced the expression levels of ALP, BMP-2, and collagen-I mRNA at the concentration of 20 μM (Figure 4). However, 1 and 5 μM treatments did not show any significant effect on the expression of osteoblastogenic factors. The effect of DHE on key osteoblastogenesis-related factors was also tested at the protein level by Western blotting. Similar to mRNA expression levels, the presence of DHE elevated the protein levels of ALP, BMP-2, collagen-I, and osteocalcin in a dose-dependent manner (Figure 5). In order to assess the details of the mechanism of action, levels of SMA/MAD homolog (Smad) and extracellular signal–regulated kinase (ERK) pathway proteins were also observed following DHE treatment. In a dose-dependent manner, DHE was able to elevate the protein levels of phosphorylated Smad1/5/8, ERK, c-Jun N-terminal kinase (JNK), and Rux-2, however it did not affect the protein levels of p-p38 significantly. In addition, DHE elevated the phosphorylated AMP-activated protein kinase (AMPK) levels while causing no change in non-activated AMPK protein levels (Figure 5).

## 3. Discussion

In the interest of determining any effect on osteoblastogenesis, DHE was tested for its potential ability to enhance osteoblast differentiation in mouse MC3T3-E1 pre-osteoblasts. As a part of differentiation, osteoblasts were observed to proliferate in a significantly elevated way. Hence, a successful osteoblastogenesis enhancer would have the same effect on differentiating osteoblasts. In this case, DHE was effective in enhancing proliferation, hence the differentiation of MC3T3-E1 pre-osteoblasts. Additionally, cell proliferation assays confirmed the non-toxic presence of DHE at the treatment concentrations.

Maturation into osteoblasts is accompanied by calcification. Accumulation of calcium is reported to be a crucial step in the formation of bone. Therefore, a successful treatment method to relieve the lack of bone formation conditions will require an efficient elevation of calcification along with ALP activity enhancement. In this regard, the effects of DHE on proliferation, ALP activity, and the mineralization of differentiating MC3T3-E1 cells were observed. Taking the increased mRNA and protein expression of osteoblastogenic transcription factors into consideration suggested that DHE from *E. cava* exerts a pro-osteoblastogenic effect on pre-osteoblasts.

Osteoblast differentiation has been studied intensively and documented in detail by several research groups. Pre-osteoblasts differentiate into mature osteoblasts through a pathway that includes specific gene and transcription factors, such as bone morphogenetic protein (BMP) 2, 4, and 7, and osteocalcin [24]. Osteocalcin is also reported to be a significant cell marker for the final differentiation state of osteoblastogenesis. BMPs are reported to elevate the expression of ALP, collagen-I, and other non-collagenous bone proteins, which indicates successful maturation into osteoblasts. Relying on reported studies, MC3T3-E1 mouse pre-osteoblasts are considered to be proper models to study in vitro osteoblast differentiation.

Results clearly suggested that DHE had a notable effect on osteoblast differentiation in a direct correlation with the sample treatment concentration. Mainly, the presence of DHE enhanced the ALP activity of differentiating osteoblasts. An elevated ALP activity is directly related to bone formation. Further, DHE was also suggested to elevate the calcification and the mRNA expression of osteogenic markers BMP-2, osteocalcin, ALP, and collagen-I.

During osteoblastogenesis, activated transmembrane serine/threonine kinase receptors follow the activation of BMPs. Activated receptors then phosphorylate the receptor Smads (R-Smads). This phosphorylation is followed by the translocation of Smads to the nucleus and regulates the transcription for osteoblastogenesis [25]. DHE was found to increase phosphorylated Smad (p-Smad) in differentiating MC3T3-E1 cells, suggesting that the effect of DHE on osteoblastogenesis may be in relation to the activation of the BMP signaling pathway. On the other hand, non-Smad pathways have also been shown to be activated in BMP-induced osteoblastogenesis [26,27]. Moreover, ERK and JNK pathways are also activated during osteoblastogenesis by BMP signaling in a Smad-independent way, along with the elevated levels of phosphorylated ERK, JNK, and Rux-2 proteins [28]. Activation of p38 protein (which plays a pivotal role in the downstream signaling of Smad-activated osteoblastogenesis) was found to be unaffected by DHE treatment. DHE was reported to inhibit the adipogenesis of 3T3-L1 through the suppression of the activation of AMPK [29]. The AMPK pathway is known to have crucial roles in energy-mediated cellular metabolisms, especially differentiation. In adipose tissue, AMPK suppression results in decreased adipogenesis through hindered activation of the peroxisome proliferator-activated receptor (PPAR) pathway. However, in terms of mesenchymal cell-precursory osteoblastogenesis, AMPK activation has a direct effect on Runx2-mediated bone formation [30,31]. DHE was able to elevate the levels of activated AMPK. Therefore, it can be suggested that DHE enhanced the osteoblastogenesis with an early interaction of the BMP signaling pathway, which has links with both Smad-dependent and Smad-independent regulation of differentiation and AMPK activation.

In conclusion, DHE was isolated from *E. cava* and is proposed as a lead compound for the possible treatment of bone-related problems where lack of bone formation is observed. DHE was shown to increase intracellular calcification, possibly with a direct enhancement of ALP activity and an intervention in the BMP signaling pathway. However, future studies to evaluate the true potential of DHE are encouraged, where its efficiency and mechanism of action on the differentiation of different cell lines (including bone marrow and mesenchymal cells) are analyzed in detail, in order to utilize DHE as a promising bone formation-enhancer. Nevertheless, DHE is evidently a potential bioactive substance for the treatment of age-related bone disorders, and is supposed to be highly active in enhancing osteoblast differentiation, indicating a promising potential to be utilized as a nutraceutical.

## 4. Materials and Methods

### 4.1. Plant Material

Leafy thalli of *E. cava* were collected along the coast of Jeju Island, Korea during the period from October 2004 to March 2005. A voucher specimen has been deposited in the author’s laboratory. The collected sample was freeze-dried and kept at −25 °C until use.

### 4.2. Extraction and Isolation

Lyophilized powder (4.0 kg) of *E. cava* was percolated in hot EtOH (3 × 10 L). The crude extract (584.3 g) was partitioned with organic solvents to yield *n*-hexane (114.3 g), CH_2_Cl_2_ (40.6 g), EtOAc (55.0 g) and *n*-BuOH (96.5 g) fractions, as well as H_2_O residue (277.9 g). The EtOAc fraction (55.0 g) of *E. cava* was subjected to column chromatography over a silica gel (silica gel 60, 0/063–0.200 mm, Merck, Kenilworth, NJ, USA) with CH_2_Cl_2_:MeOH (30:1 to 1:1), yielding 16 sub-fractions (EF01 to EF16). Sephadex LH-20 (bead size 25–100 μm, Sigma, St. Louis, MO, USA) column chromatography of fraction 2 (EF02, 1.1 g) was conducted with MeOH, yielding five sub-fractions (EF0201 to EF0205). Sephadex LH-20 column chromatography of fraction 2 (EF0202, 110.4 mg) using identical solvent conditions led to the isolation of dioxinodehydroeckol (88.0 mg). The structural identity of dioxinodehydroeckol (DHE) was verified by comparison with published spectral data.

#### Dioxinodehydroeckol (DHE)

^1^H-NMR (400 MHz, DMSO-*d*_6_) δ: 9.77 (1-OH), 9.64 (9-OH), 9.60 (6-OH), 9.27 (3-OH), 9.26 (11-OH), 6.10 (1H, s, H-7), 6.04 (1H, d, *J* = 2.7 Hz, H-2), 6.01 (1H, d, *J* = 2.7 Hz, H-10), 5.84 (1H, d, *J* = 2.7 Hz, H-4), 5.82 (1H, d, *J* = 2.7 Hz, H-12); ^13^C-NMR (100 MHz, DMSO-*d*_6_) δ: 153.3 (C-3), 153.0 (C-11), 146.1 (C-1), 146.0 (C-9), 142.1 (C-4a), 141.7 (C-12a), 140.1 (C-6), 137.2 (C-7a), 131.6 (C-13b), 125.9 (C-5a), 122.7 (C-8a), 122.5 (C-13a), 122.3 (C-14a), 98.8 (C-2, 10), 97.6 (C-7), 93.9 (C-4, 12).

### 4.3. Cell Culture and Osteoblast Differentiation

Murine osteoblast-like MC3T3-E1 cells were seeded in six-well plates at a density of 1 × 10^5^ cells/well and grown to confluence in α-Modified minimal essential medium (αMEM) supplemented with 10% heat-inactivated fetal bovine serum (FBS), 1 mM sodium pyruvate, 100 units/L penicillin, and 100 mg/L streptomycin at 37 °C in a humidified atmosphere of 5% CO_2_. At confluence, the cells were cultured for osteoblast differentiation with culture medium containing 50 μg/mL ascorbic acid and 10 mM β-glycerophosphate. After three days, the DHE from *E. cava* was administered to the culture medium and further incubated for four days. DHE was administered to cell culture with each medium change, staring from day three of incubation until the cell harvest (day seven) for further experiments.

### 4.4. Cell Proliferation Assay

The effect of the DHE from *E. cava* on the proliferation of MC3T3-E1 osteoblasts was measured by 3-(4,5-dimethylthiazol-2-yl)-2,5-diphenyltetrazolium bromide (MTT) assay. Briefly, cells were grown in 96-well plates at a density of 5 × 10^3^ cells per well for 24 h. Cell differentiation was induced with culture medium containing 50 μg/mL ascorbic acid and 10 mM β-glycerophosphate. After three days, the cells were treated with DHE from *E. cava* for four days. Then, the cells were incubated with 100 μL of MTT reagent (1 mg/mL) for 4 h. Finally, 100 μL DMSO was added to dissolve the formazan salt, and cell proliferation was measured by the absorbance at 540 nm using a microplate reader (Tecan Austria GmbH, Grodig, Austria).

### 4.5. Cellular Alkaline Phosphatase (ALP) Activity

Cellular ALP activity was measured in MC3T3-E1 cells treated with DHE from *E. cava* and in control cells incubated for three days. The cell monolayer was gently washed twice with PBS and lysed using 0.1% Triton X-100 and 25 mM Carbonate buffer. The lysates were centrifuged at 4 °C 12,000× *g* for 15 min. The supernatants were used to measure the ALP activity using enzyme assay buffer (15 mM ρ-nitrophenyl phosphate, 1.5 mM MgCl_2_, and 200 mM carbonate buffer). The absorbance of the reactive solution was measured at 405 nm.

### 4.6. Alizarin Red Staining

For Alizarin Red staining, cells were fixed in 70% ice-cold ethanol for 30 min, washed with distilled H_2_O and stained with filtered alizarin red solution for 10 min. After staining, the alizarin red staining solution was removed, and the plates were rinsed five times with distilled water. Images of mineralization in MC3T3-E1 osteoblasts were collected by an Olympus microscope (Tokyo, Japan). Finally, the dye retained in the cells was eluted with 10% cetylpyridinium chloride (Sigma, St. Louis, MO, USA) solution and quantified by measuring optical absorbance at 560 nm using a microplate reader (Bio-Tec instrument, San Diego, CA, USA).

### 4.7. RNA Extraction and Reverse Transcription-Polymerase Chain Reaction Analysis

Total RNA was isolated from MC3T3-E1 osteoblasts treated with/without DHE using Trizol reagent (Invitrogen Co., Carlsbad, CA, USA). For synthesis of cDNA, RNA (2 μg) was added to RNase-free water and oligo (dT), denatured at 70 °C for 5 min and cooled immediately. RNA was reverse transcribed in a master mix containing 1× RT buffer, 1 mM dNTPs, 500 ng oligo (dT), 140 U M-MLV reserve transcriptase, and 40 U RNase inhibitor at 42 °C for 60 min and at 72 °C for 5 min using an automatic T100 Thermo Cycler (Bio-Rad, Hercules, CA, USA). The target cDNA was amplified using the following sense and antisense primers: forward 5′-CCA-GCA-GGT-TTC-TCT-CTT-GG-3′ and reverse 5′-CTG-GGA-GTC-TCA-TCC-TGA-GC-3′ for ALP; forward 5′-GGA-CCC-GCT-GTC-TTC-TAG-TG-3′ and reverse 5′-GCC-TGC-GGT-ACA-GAT-CTA-GC-3′ for BMP-2; forward 5′-GCT-GTG-TTG-GAA-ACG-GAG-TT-3′ and reverse 5′-CAT-GTG-GGT-TCT-GAC-TGG-TG-3′ for Osteocalcin; forward 5′-GAG-CGG-AGA-GTA-CTG-GAT-CG-3′ and reverse 5′-TAC-TCG-AAC-GGG-AAT-CCA-TC-3′ for Collagen I; forward 5′-CCA-CAG-CTG-AGA-GGG-AAA-TC-3′ and reverse 5′-AAG-GAA-GGC-TGG-AAA-AGA-GC-3′ for β-actin. The amplification cycles were carried out at 95 °C for 45 s, 60 °C for 1 min, and 72 °C for 45 s. After 30 cycles, the PCR products were separated by electrophoresis on 1.5% agarose gel for 30 min at 100 V. Gels were then stained with 1 mg/mL ethidium bromide visualized by UV light using Davinch-Chemi imager™ (CAS-400SM, Davinch-K, Seoul, Korea).

### 4.8. Western Blot Analysis

Western blotting was performed according to standard procedures. Briefly, cells were lysed in RIPA lysis buffer (Sigma-Aldrich Corp., St. Louis, MO, USA) at 4 °C for 30 min. Cell lysates (35 μg) were separated by 12% SDS-polyacrylamide gel electrophoresis, transferred onto a polyvinylidene fluoride membrane (Amersham Pharmacia Biotech., England, UK), blocked with 5% skim milk, and hybridized with primary antibodies (diluted 1:1000) against ALP, BMP-2, collagen-1, osteocalcin, p-Smad1/5/8, Rux-2, p-ERK, p-JNK, p-p38, AMPK, p-AMPK, and β-actin. After incubation with horseradish peroxidase-conjugated secondary antibody at room temperature, immunoreactive proteins were detected using an electrochemiluminescence (ECL) assay kit (Amersham Pharmacia Biosciences, England, UK) according to the manufacturer’s instructions. Western blot bands were visualized using a Davinch-Chemi imager™ (CAS-400SM, Davinch-K, Seoul, Korea).

### 4.9. Statistical Analysis

The data were presented as mean ± SD. Differences between the means of the individual groups were analyzed using the analysis of variance (ANOVA) procedure of Statistical Analysis System, SAS v9.1 (SAS Institute, Cary, NC, USA) with Duncan’s multiple range tests. The significance of differences was defined at the *p* < 0.05 level.
